# Eugenics

**Published:** 1914-01

**Authors:** 


### EDITORIAL

The Business Office of the *Journal-Record* is 23 West Alabama Street.

The Editorial Office is 1014-15 Atlanta National Bank Building.

Address all Business Communications to *Journal-Record of Medicine*, 23 West Alabama Street.

Make remittances payable to *The Journal-Record of Medicine.*

On matters pertaining to the Editorial and Original Communications, address Edgar G. Ballenger, M. D., Atlanta, Ga.

Reprints of Original Articles will be furnished at cost price. Requests for the same should always be made in THE MANUSCRIPT.

We will present, postpaid, on request to each contributor of an original article, twenty (20) marked copies of *The Journal-Record of Medicine* containing such article.

### EUGENICS.

A healthy birth and a normal start in life is an ideal that appeals to every fair-minded person as the right of every child. Even those who are diseased do not wish to transit suffering to their offspring and if marriage has occurred there are efforts toward the suppression of conception which are expected to prevent ailing children coming to birth.

Once the idea of eugenics has come into public thought, enthusiasm is aroused and people try to do in a day what has been largely undone in several thousand years. They turn to law as if it were something that could change people in a twinkling of an eye and make them new and different.

Law is one of two things. It is something to be revered and obeyed because it is the crystalized expression of what the people find is best for them in conduct, or it is something to be rebelled against, evaded, disobeyed and generally condemned, because it imposes what is not understood and demands changes that are obnoxious in their extent.

The first kind of law brings government into respect; the second brings government and the legislators into contempt.

We are witnessing just now the failure of a sumptuary law to carry out its provisions because they were made enthusiastically instead of wisely. We see the contempt of the people in the advertisement of certain officials in Wisconsin to marry couples without any eugenic certificates for ten cents which is the simple cost of registration of the marriage. We read that the number of weddings has fallen off marvelously and that thus public policy is interfered with. We are told that despite the law that makes it a felony for couples to leave the state to marry, the same thing is done and that families are beginning their career with the shadow of conviction and imprisonment hanging over them. It is due to the bad law, the kind that says you "must" to every one and not "this is what the majority of us have been doing for a long time and therefore everyone shall do it."

People dislike being ordered about for their own good and physicians know this perhaps better than any other set of men. Quarantine regulations show this every day and many a family doctor has had to struggle to retain his hold upon life long patients because he has declined to be party to the evasion of a law that will save people's lives.

Prohibition as a law has failed, for every man knows that there is but slight difficulty in getting the prohibited material almost anywhere in the district. Moreover, the liquor reports show that there has been little or no falling off of the production of the prohibited material in the country at large.

Laws against vice merely change the aspect of it no matter how largely Mayors and Chiefs of Police may proclaim the spotless purity of their domains.

We can not do by law what the people do not want to do. Everyone knows this and has said it many times over. Yet enthusiasm here and a sop to conscience there have allowed laws sumptuary in character to go on the statute books, and eugenic legislation is one of them.

You may say that only by imperious laws was the Panama Zone freed from malaria and Cuba and New Orleans purified from yellow fever. And in some measure that may be an exception to the rule. But the area in each case was comparatively small and the active enemy elimited. Gorgas knew the mosquito he was to eliminate and he understood thoroughly the enteric germs he was to keep from the workmen. Lazear gave his life to prove that a certain mosquito carried yellow fever. These insects were tangible, their habits of life were known, they were get-at-able, and their resulting destruction proved the value of the work. But even now with all this before them, men in communities that have not suffered with malaria will not take the warnings that are given them as to the probable infection of the mosquitoes and subsequently of themselves. They will not drain their pools, no matter what influence is brought to bear upon them until death has had its toll and a public sentiment has been established.

In the diseases that eugenic law seeks to prevent, the germs have no such tangible character as the mosquito gives. Syphilis and Gonorrhoea have habitats that can not be invaded no matter what the authority nor how strenuous the hand that would seek them. Sexual intercourse can not be stopped, nay, scarcely regulated no matter what the law nor the prophets. The diseases clinging thereto can not be legislated away to the extent of a single germ nor can Sheriffs and Marshals, be they laymen or physicians apprehend a single spirochete or coccus.

The same is true of Tuberculosis but not in so great a measure. One could imagine the discovery of nearly all the tubercular cases in a community and their subsequent isolation, but it would be a Utopain and impractical dream. The biggest club in the world swung with the greatest possible force might kill a few million bacilli, but the breeze of its passage would gently waft millions more to the breathing apparatus of the waiting thousands of spectators or innocent passers by.

These diseases can not be controlled by law nor can their presence be ascertained with any degree of certitude in an examination for a marriage license unless they are in very pronounced stages when an examination would not be necessary. They can not be ascertained, that is, except with a very exhaustive examination that might take months to complete and which is impracticable to everyone except some over enthusiastic law makers.

What then is the solution? Eugenics should be taught to young parents who have children coming along to demonstrate its principles, and these children should be taught the fundamentals of the sex question by the parents from the time they ask the first questions till they have mastered it and entered into the control of their sexual life.

If the children know these things as they are, there is no need to fear immodesty or lack of purity. Ignorance is not innocence. Tell them the truth so that they can understand it and all will be well. Then when these instructed ones come to marry, they will be clean themselves and seek mates among the clean. Eventually eugenics will be an accomplished fact and no laws are needed. Heredity will exhaust itself so far as disease is concerned in a few generations if the individuals are instructed and made wise as they develop.

Who is to teach these things? It should be a matter of public discussion in the papers and on the forum until parents learn that there is such a subject and that it is of importance to them. Then every physician stands ready to tell every family under his care all they want to know about the matter and to guide them from time to time in the proper instruction of the children. Once an intelligent interest is established there will be no trouble about it and the teaching will never be sensational nor tend to crime and lubricity as at present. It takes time and there is where it is sensible. We can not have Eugenics in one generation.

